# HIV/AIDS Mortality Trends in Lang Son, Vietnam: Insights from a Population-Based Mortality Registration from 2005 to 2018

**DOI:** 10.3390/tropicalmed10020052

**Published:** 2025-02-10

**Authors:** Ngoan Tran Le, Linh Thuy Le, Ngan Dieu Thi Ta, Hung Manh Nguyen, Toan Ha

**Affiliations:** 1 Institute of Research and Development, Duy Tan University, Da Nang City 550000, Vietnam; 2 Department of Occupational Health, Institute of Preventive Medicine and Public Health, Hanoi Medical University, Hanoi City 100000, Vietnam; 3 Laboratory of Embryology and Genetics of Human Malformation, Imagine Institute, INSERM, UMR, 1163 Paris, France; lelinh2611@gmail.com; 4 Department of Infectious Diseases, Hanoi Medical University, Hanoi City 100000, Vietnam; tadieungan@hmu.edu.vn; 5 Faculty of Medicine, University of Medicine and Pharmacy, Ho Chi Minh City 700000, Vietnam; manhhungng.97@gmail.com; 6 Department of Infectious Disease and Microbiology, School of Public Health, University of Pittsburgh, Pittsburgh, PA 15261, USA

**Keywords:** HIV/AIDS, mortality, gender, Lang Son Province, Vietnam

## Abstract

The HIV epidemic remains a major public health issue globally and in Vietnam. This study assesses changes in HIV/AIDS-related mortality rates over time in Lang Son Province, Vietnam, from 2005–2018. We performed a descriptive epidemiological study using a population-based mortality registration system to examine HIV/AIDS-related mortality. HIV/AIDS-related mortality was converted to a crude and adjusted rate per 100,000 person-years using the World Health Organization’s standard population for 2000–2025. The mortality rate ratio and 95% confidence interval were estimated to examine the province’s time trend from 2005 to 2018. The adjusted mortality rate for HIV/AIDS in Lang Son Province was 12.3 and 2.4 per 100,000 for men and women, respectively, with a male-to-female ratio of 5.1. The province experienced a 94% reduction in HIV/AIDS-related deaths between 2005 and 2018. The mortality rate ratio for 2018 compared to 2005 was lower for men (0.056, 95% CI: 0.029, 0.110) than for women (0.080, 95% CI: 0.019, 0.338). The findings show a gradual decline in HIV/AIDS-related mortality rates in Lang Son Province, Vietnam. However, significant gender disparities in mortality remain a major concern, and HIV remains a significant burden. This highlights the urgency for major efforts to prevent HIV transmission and address these disparities to effectively end the HIV epidemic in Lang Son and throughout Vietnam.

## 1. Introduction

Despite remarkable global progress in HIV prevention and treatment, HIV remains a major public health challenge throughout the world. Globally, in 2023 alone, an estimated 1.3 million people newly acquired HIV, bringing the estimated global total of people living with HIV (PLWH) to 39.9 million [1]. Furthermore, approximately 630,000 individuals died from AIDS-related illnesses that same year. The global community has committed to ending the HIV/AIDS epidemic by 2030 under Sustainable Development Goal 3 [2]. However, significant challenges and barriers continue to hinder the fight against the HIV/AIDS epidemic [3]. PLWH and those at risk for HIV continue to lack access to essential prevention, treatment, and care services, especially in low- and middle-income countries [3]. HIV-related stigma and discrimination remain prevalent barriers to HIV testing, disclosure, and healthcare access, hindering timely diagnosis and engagement in care [4,5].

Vietnam has made significant progress in HIV control, with substantial declines in new infections since 2010. The prevalence of HIV has decreased from 0.4% in 2010 to a stable 0.3% since 2015 [6,7]; however, HIV remains a serious public health concern. In 2023, Vietnam reported 6100 new HIV infections and 4100 AIDS-related deaths [6]. However, disparities in HIV prevalence remain challenging, with higher rates observed among people who inject drugs (PWID), men who have sex (MSM) with men, and female sex workers (FSW) [6]. The HIV prevalence among MSM, PWID, and FSW was 12.5%, 9.1%, and 2.5%, respectively, in 2023 [6].

The Law on HIV/AIDS Prevention and Control was passed by the National Assembly of Vietnam in 2006. This legislation established the HIV/AIDS Prevention and Control Department under the Ministry of Health, which oversees a four-tiered hierarchical healthcare system comprising provincial, district, and commune healthcare levels. The system includes 10,769 commune health stations (CHSs), which facilitate comprehensive data collection on HIV testing, new infections, prevalence, and mortality. The results are then shared throughout this four-level system. Each HIV/AIDS related death case is compared with the patient’s records at the CHS to determine the underlying cause of death. As a result, the information on HIV/AIDS-related mortality collected by CHSs is considered reasonably reliable. This reliability is supported by a previous study in Vietnam, which found that the reporting of HIV/AIDS-related deaths demonstrated a moderate level of accuracy and reliability, with kappa scores ranging from 0.57 to 0.69 (*p* < 0.01) [8]. While nationwide HIV/AIDS deaths were reported as 4674 in 2005, 5190 in 2006, and 5569 in 2007, respectively [9], these figures lacked provincial disaggregation and geographic mapping, hindering effectively targeted prevention efforts.

**Figure 1:**
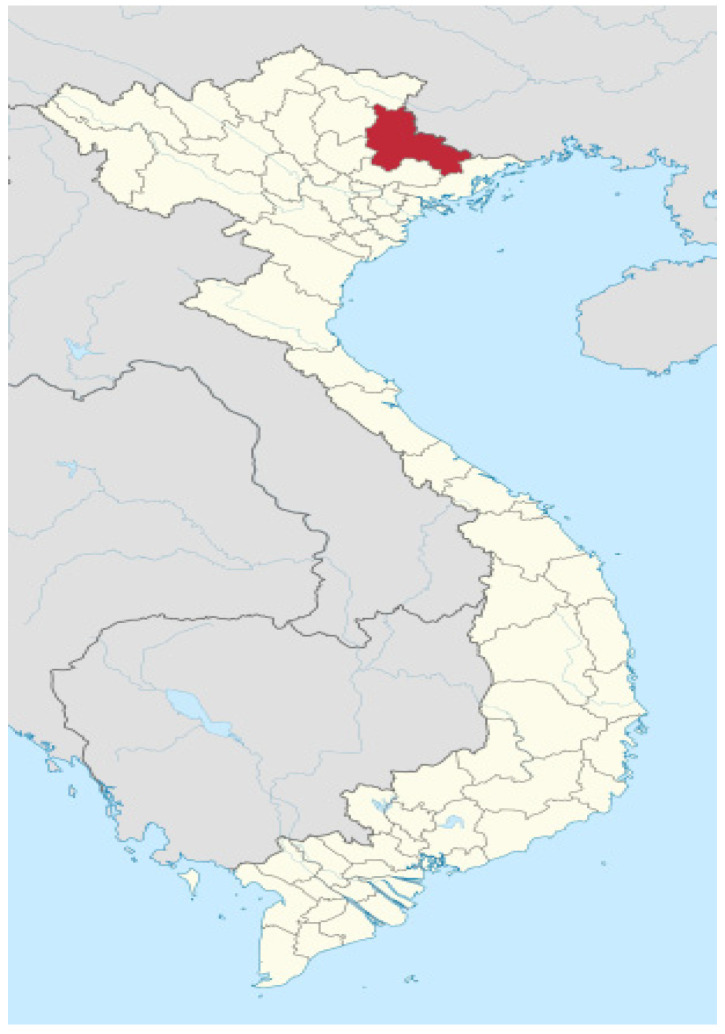
Lang Son Province (Red), Vietnam.

Lang Son, a mountainous province in Northeast Vietnam, covers a natural area of 8187.25 km^2^ and borders China’s Guangxi Province (Figure 1). The province’s population in 2019 was 782,811, was primarily rural (80.7%), and included a high proportion of ethnic minorities (84.74%). There were 30,583 poor households (15.83%) and 21,267 near-poor households (11.01%). Given these geographic and economic conditions, Lang Son represents the mountainous regions in Vietnam, with limited access to high-quality healthcare. However, the province’s proximity to Ning Ming County, Guangxi Province, China, facilitates substantial cross-border trade, employment, and, unfortunately, drug trafficking and sex work [10]. These activities increase the likelihood of sexual risk behaviors and vulnerability to HIV/AIDS. Understanding the mortality trends in Lang Son can provide valuable insights into addressing the challenges faced by similar regions in Vietnam.

As of the end of 2014, Lang Son Province had recorded a total of 3397 HIV cases since the first case was detected in 1993. Of these, 1973 cases progressed to AIDS, and 1808 of those 1973 individuals who progressed to AIDS died from the disease [11]. Despite a significant decline in HIV/AIDS cases between 2009 and 2014, the province continues to ineffectively address the disease, partly due to the lack of comprehensive data on HIV-related mortality. To address this gap, this study assessed HIV/AIDS-related mortality trends in Lang Son Province, Vietnam, from 2005 to 2018 to provide evidence for informing targeted interventions. This will help ensure that the province can effectively prevent and control the spread of HIV, reducing the impact of HIV/AIDS on the population of Lang Son Province and contributing to ending the HIV epidemic in Vietnam.

## 2. Materials and Methods

### 2.1. Study Design and Data Source

This descriptive epidemiological study used a population-based mortality registration system to examine HIV/AIDS-related mortality trends in Lang Son Province from 2005 to 2018. Data were collected using the A6 mortality system, an official death recording system managed by the Lang Son Center for Disease Control and Prevention (CDC). The A6 mortality systems were validated and presented as reliable and feasible systems for mortality recording. These unique mortality reporting systems were introduced nationwide in Vietnam in 1992 and have been previously validated to achieve good sensitivity, specificity, and accuracy [9,12,13,14,15].

All deaths within communities were systematically recorded at local commune health stations (CHSs) using the A6 form. Health station heads compiled and verified data monthly, which were submitted annually to the Lang Son CDC. This process created a comprehensive mortality database from 2005 onward, encompassing case ID, age, sex, date, place, cause of death, and ICD-10 code.

The Lang Son population-based mortality registration system encompassed over 226 state commune health stations across 11 cities and districts in the province. The Lang Son CDC collected annual data from the population served by each state commune health station. To ensure the accuracy and completeness of the records, the head of each commune health station provided training to medical workers in the meticulous tracking and follow-up of each morbidity case for at least six months, ensuring that the outcome for each resident was accurately identified. As a result, all recorded deaths included well-documented causes based on medical records. Population data were rigorously cross-checked against multiple independent sources, including the provincial statistics department. Eligible cases for this study included all HIV/AIDS-related deaths recorded from January 2005 to December 2018 in Lang Son Province, resulting in a total of 707 HIV/AIDS-related deaths. Cases involving individuals from China or other provinces who were temporarily residing in Lang Son were excluded.

### 2.2. Data Analysis

Data were reviewed and cross-checked between information sources, cleaned, encoded, reported using Excel software, and analyzed by STATA 15.0. The crude mortality rates were calculated separately for men and women by dividing the number of HIV/AIDS-related deaths by the respective total population and then multiplying by 100,000. Age-standardized mortality rates (ASRs) were estimated using the World Health Organization (WHO) standard population for 2000–2025 [16]. Mortality rate ratios (MRR) and their corresponding 95% confidence intervals (95%CI) were calculated to assess trends in HIV/AIDS mortality from 2005 to 2018, adjusting for age groups (0–9, 10–19, 20–29, 30–39, 40–49, 50–59, 60–69, 70–79, 80+) and sex.

There were no available data on HIV/AIDS-related deaths for 2009 and 2010. The reason for missing data from 2009 and 2010 for the whole province was that we lost each state commune health station’s completed mortality registration paper forms. The reference group was 2005, and the mortality rates ratio and 95% confidence interval were estimated for 2006–2008 and 2011–2018 only. We have checked for missing data by year and for each state commune health station. Only available data on HIV/AIDS mortality and the population denominator by year and by each state commune health station were pooled into the data for the whole province for final data analysis. Since the number of cases from 2015 to 2018 was fewer than 20 for both men and women combined, we did not conduct an analysis by rural subpopulation or age group.

## 3. Results

### 3.1. HIV/AIDS-Related Mortality

Table 1 presents the mortality data related to HIV using ICD codes B20-B24 in Lang Son Province from 2005 to 2018, categorized by sex. During the study period, there were a total of 707 HIV/AIDS-related deaths, with an overall age-standardized rate (ASR) of 6.6 per 100,000 person-years, which accounts for 1.44% of all deaths in the province (out of a total of 49,253 deaths). Among men, 594 HIV-related deaths were recorded, resulting in an ASR of 10.8 per 100,000 person-years. In contrast, women accounted for 113 HIV/AIDS-related deaths, corresponding to an ASR of 2.2 per 100,000 person-years. The male-to-female ratio for age-standardized mortality was 5.1, indicating a significantly higher mortality rate among men compared to women. Notably, all deaths occurred in individuals under 70 years of age.

### 3.2. HIV/AIDS-Related Mortality by Gender

Table 2 provides a year-by-year breakdown of HIV/AIDS-related deaths for both genders from 2005 to 2018 in Lang Son Province. In 2005, the ASR was 22.3 per 100,000 person-years. Over time, the rates steadily declined, reaching an ASR of 1.3 per 100,000 person-years by 2018. For men, HIV/AIDS-related mortality decreased significantly from an ASR of 38.0 per 100,000 person-years in 2005 to 2.2 in 2018, with a 94.4% reduction in the MRR (0.056, 95% CI: 0.029, 0.110). For women, the ASR dropped from 6.3 per 100,000 person-years in 2005 to 0.5 in 2018, reflecting a 92.0% decrease in the MRR (0.080, 95% CI: 0.019, 0.338).

### 3.3. HIV/AIDS-Related Age-Specific Mortality Rates

**Figure 2:**
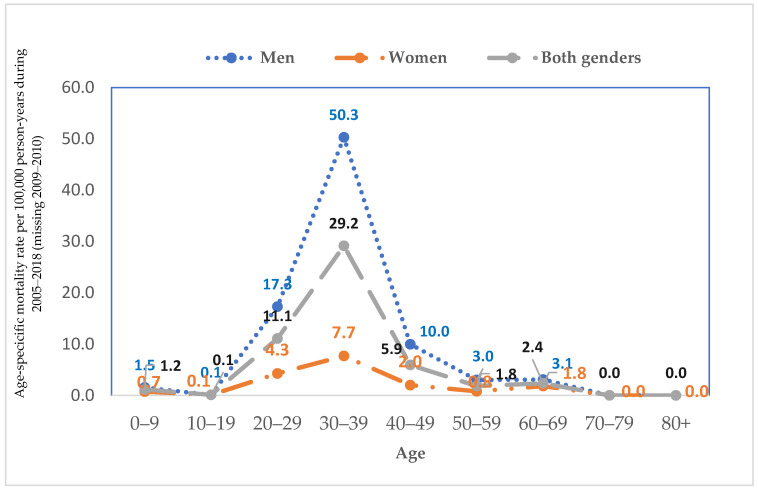
Age–specific mortality rates for men, women, and both genders combined from 2005 to 2018 in Lang Son Province, Vietnam.

Figure 2 shows age-specific mortality rates per 100,000 person-years, stratified by gender from 2005 to 2018 (excluding 2009–2010). For both genders combined, the mortality rate peaks in the 30–39 age group at 29.2, followed by the 20–29 group at 11.1. Men showed a significantly higher mortality rate, with a peak at 50.3 in the 30–39 age group, followed by 17.3 in the 20–29 age group and 10.0 in the 40–49 group. In contrast, women exhibit much lower mortality rates, with a peak of 7.7 in the 30–39 age group and 4.3 in the 20–29 group. Notably, both genders exhibit minimal mortality rates in the youngest (0–9) and oldest age categories (70+).

## 4. Discussion

The study results demonstrated a consistent and significant decline in HIV/AIDS-related mortality for both genders in Lang Son Province from 2005 to 2018. The ASR dropped dramatically from 22.3 to 1.3 per 100,000 person-years during this period, although the magnitude and timing of this decline varied between sexes. This substantial reduction indicates the province’s progress in combating HIV/AIDS, likely attributable to improved access to ART, enhanced prevention efforts, and increased public awareness, which have been documented in various studies [17,18,19]. These findings highlight the effectiveness of public health interventions in reducing HIV/AIDS mortality in Lang Son Province.

However, persistent gender disparities in mortality rates necessitate continued efforts to sustain the downward trend and address these inequalities. The data reveal a significant gender disparity in HIV-related mortality, with men experiencing a much higher death rate compared to women (10.8 vs. 2.2 per 100,000 person-years). This male-to-female ratio of 5.1 underscores the stark differences in HIV-related mortality between men and women in Lang Son Province, with men bearing a significantly higher mortality burden. Previous studies in the US [20], Brazil [21], China [22], and Iran [23] have also reported higher mortality rates among men compared to women. For instance, research in China showed that the rate of HIV/AIDS mortality increased more quickly in men than in women from 1990 to 2016, with HIV/AIDS mortality in men being three times higher than that in women in 2016 [22]. The study in Iran also found that males exhibited a 3.20-fold higher mortality rate than females [23]. In contrast, a study in Ethiopia revealed that HIV-related deaths are disproportionately higher among women than men [24].

The majority of HIV/AIDS-related deaths occurred among individuals aged 20–39 years, emphasizing the disproportionate impact of the epidemic on younger populations. This underscores the critical need to focus large-scale HIV primary prevention efforts on this age group, which faces a higher risk and mortality rate, peaking at 50.3 per 100,000 for men and 7.7 per 100,000 for women in the 30–39 age group. This finding aligns with global trends in which younger adults, particularly men aged 30–39, exhibit the highest mortality rates [25]. A systematic analysis of the Global Burden of Diseases study revealed that between 2007 and 2017, the highest percentage of HIV deaths occurred among females aged 30–34 and males aged 35–39 [25]. Early intervention strategies, including comprehensive sexual health education, accessible testing, and timely treatment, are essential to curb the spread of HIV/AIDS and mitigate its devastating consequences within this vulnerable population. Moreover, ensuring that accessible testing services are available is vital.

The data reveals a consistent and substantial decline in HIV/AIDS mortality rates for both men and women. While both sexes experienced significant reductions, men demonstrated a slightly steeper decline, with a 94.4% decrease compared to a 92.0% decrease for women. This gender disparity in mortality reduction suggests underlying factors influencing the effectiveness of HIV interventions. Potential contributors to this difference may include disparities in treatment adherence, lack of communication between women and healthcare providers, access to healthcare, and stigma [26,27,28,29]. For instance, research indicates that women often face unique barriers to accessing HIV care, including gender-related stigma and discrimination within healthcare settings, which can hinder their engagement in treatment [26]. A study in the US revealed that poverty, unemployment, housing insecurity, and transportation needs were identified as the primary structural obstacles women face in maintaining consistent engagement in HIV care [28]. Further research is needed to explore the factors associated with the reduction in AIDS-related mortality among men and women in Lang Son.

This study has several limitations. The reliance on the A6 mortality registration system may have introduced potential biases due to the underreporting or misclassification of deaths. Additionally, the analysis was conducted at a group level rather than an individual level, which limits the ability to draw definitive conclusions about individual-level risk and protective factors associated with HIV/AIDS mortality. While the data offer valuable insights, further research is needed to explore the underlying causes of gender disparities, the role of co-morbidities, and the long-term impact of HIV interventions across different demographic groups. Qualitative studies could also provide a deeper context regarding the barriers and facilitators to accessing HIV treatment and care in Lang Son Province. Finally, the unavailability of data on HIV/AIDS-related deaths for 2009 and 2010 may have some impact on the overall results. However, the consistent mortality trend from 2005 to 2018 suggests that the data for these years were unlikely to have significantly deviated from the overall pattern.

## 5. Conclusions

This study provides valuable insights into trends of HIV/AIDS-related mortality and gender disparities over a 13-year period in Lang Son Province, Vietnam. The findings show a gradual decline in mortality rates. However, gender disparities and the ongoing burden of the disease underscores the need for major efforts to prevent HIV transmission and address these disparities. Future research should focus on identifying specific factors contributing to higher mortality rates among men and evaluating the effectiveness of different prevention and treatment interventions in reducing HIV/AIDS-related mortality. By addressing these issues, public health program officials can develop targeted interventions and programs to further reduce the impact of HIV/AIDS in the province, ultimately contributing to the ending of HIV epidemic, in Vietnam and globally, by 2030.

## Figures and Tables

**Table 1:** HIV/AIDS-related mortality by gender during 2005–2018 in Lang Son Province.

Gender	Year	Cause	Total	Crude Rate ^&^	ASR ^@^	WHO ^$^
Men	2005–2018	B20-B24	594	13.2	10.8	12.3
Women	2005–2018	B20-B24	113	2.5	2.2	2.4
Both genders	2005–2018	B20-B24	707	7.8	6.6	7.4

^&^ Crude rate per 100,000 person-years; ^$^ age-standardized rate per 100,000 person-years using the World Health Organization standard population for 2000–2025 (WHO); ^@^ age-standardized rate per 100,000 person-years using the SEGI world standard population (in the 1960s, ASR). Men to women ratio (WHO standard population) = 5.1 (12.3/2.4). HIV: human immunodeficiency virus; AIDS: acquired immunodeficiency syndrome.

**Table 2:** Mortality due to HIV/AIDS by gender and year from 2005 to 2018 in Lang Son Province.

Year	Case	Crude Rate ^&^	WHO ^$^	MRR (95%CI) ^$$^	*p*
**Both genders**					
2005	170	23.6	22.3	1 (reference)	
2006	153	20.9	19.6	0.885 (0.711, 1.101)	0.27
2007	117	15.9	15.2	0.673 (0.532, 0.852)	0.001
2008	98	13.1	12.6	0.556 (0.434, 0.713)	<0.001
2011	38	5.2	5.1	0.219 (0.154, 0.312)	<0.001
2012	27	3.6	3.4	0.153 (0.102, 0.230)	<0.001
2013	22	3.0	2.9	0.126 (0.081, 0.197)	<0.001
2014	27	3.5	3.3	0.146 (0.097, 0.220)	<0.001
2015	16	2.0	1.9	0.086 (0.052, 0.144)	<0.001
2016	13	1.7	1.5	0.070 (0.040, 0.123)	<0.001
2017	15	1.9	1.8	0.080 (0.047, 0.135)	<0.001
2018	11	1.4	1.3	0.059 (0.032, 0.109)	<0.001
**Men**					
2005	147	41.1	38.0	1 (reference)	
2006	130	35.7	32.9	0.869 (0.687, 1.100)	0.24
2007	103	28.2	26.0	0.685 (0.533, 0.882)	0.003
2008	80	21.9	20.4	0.525 (0.400, 0.689)	<0.001
2011	27	7.4	7.1	0.180 (0.120, 0.272)	<0.001
2012	21	5.7	5.3	0.138 (0.087, 0.217)	<0.001
2013	17	4.6	4.5	0.113 (0.068, 0.186)	<0.001
2014	25	6.4	6.1	0.157 (0.102, 0.239)	<0.001
2015	15	3.8	3.7	0.093 (0.055, 0.159)	<0.001
2016	9	2.3	2.1	0.056 (0.029, 0.110)	<0.001
2017	11	2.8	2.6	0.068 (0.037, 0.125)	<0.001
2018	9	2.3	2.2	0.056 (0.029, 0.110)	<0.001
**Women**					
2005	23	6.4	6.3	1 (reference)	
2006	23	6.3	5.9	0.983 (0.552, 1.753)	0.95
2007	14	3.8	3.9	0.596 (0.306, 1.157)	0.13
2008	18	4.8	4.7	0.755 (0.407, 1.399)	0.37
2011	11	3.0	3.0	0.469 (0.229, 0.963)	0.039
2012	6	1.6	1.5	0.251 (0.102, 0.617)	0.003
2013	5	1.3	1.3	0.212 (0.081, 0.558)	0.002
2014	2	0.5	0.5	0.080 (0.019, 0.340)	0.001
2015	1	0.3	0.2	0.040 (0.005, 0.294)	0.002
2016	4	1.0	1.0	0.159 (0.055, 0.461)	0.001
2017	4	1.0	1.1	0.157 (0.054, 0.455)	0.001
2018	2	0.5	0.5	0.080 (0.019, 0.338)	0.001

^&^ Crude rate per 100,000 person-years; ^$^ age-standardized rate per 100,000 person-years using the World Health Organization standard population for 2000–2025 (WHO); ^$$^ MRR (95%CI): Mortality rate ratios and corresponding 95% confidence intervals, adjusting for age groups (0–9, 10–19, 20–29, 30–39, 40–49, 50–59, 60–69, 70–79, 80+) and sex when applicable.

## Data Availability

The data that support the findings of this study are available on request from the corresponding author.

## Abbreviations

The following abbreviations are used in this manuscript:

AIDS	acquired immunodeficiency syndrome.
ASRs	age-standardized mortality rates
CDC	Center for Disease Control and Prevention
CHSs	commune health stations
MRR	mortality rate ratios
PLWH	people living with HIV
WHO	World Health Organization

## References

[1] UNAIDS AIDS by the Number. https://www.unaids.org/en.

[2] United Nations Transforming Our World: The 2030 Agenda for Sustainable Development. Department of Economic and Social Affairs. https://sustainabledevelopment.un.org/post2015/transformingourworld/publication.

[3] Dombrowski J.C., Simoni J.M., Katz D.A., Golden M.R. (2015). Barriers to HIV Care and Treatment Among Participants in a Public Health HIV Care Relinkage Program. AIDS Patient Care STDS.

[4] Martinez J., Harper G., Carleton R.A., Hosek S., Bojan K., Clum G., Ellen J. (2012). The impact of stigma on medication adherence among HIV-positive adolescent and young adult females and the moderating effects of coping and satisfaction with health care. AIDS Patient Care STDS.

[5] Pellowski J.A. (2013). Barriers to care for rural people living with HIV: A review of domestic research and health care models. J. Assoc. Nurses AIDS Care.

[6] UNAIDS (2023). Country-Viet Nam. https://www.unaids.org/en/regionscountries/countries/vietnam.

[7] Socialist Republic of Vietnam (2015). Contry Report-15 Years Achieving the Viet Nam Millennium Development Goals. https://vietnam.un.org/sites/default/files/2019-08/Bao%20cao%20TIENG%20ANH%20-%20MDG%202015_trinh%20TTCP.pdf.

[8] Hong T.T., Phuong Hoa N., Walker S.M., Hill P.S., Rao C. (2018). Completeness and reliability of mortality data in Viet Nam: Implications for the national routine health management information system. PLoS ONE.

[9] Le N.T., Nguyen T.V., Nguyen H.T., Ikeda S. (2020). Mortality due to HIV/AIDS in Viet Nam: Time trend and related socio-economic status in some populations and periods from 2005 to 2014. AIDS Care.

[10] Des Jarlais D.C., Johnston P., Friedmann P., Kling R., Liu W., Ngu D., Chen Y., Hoang T.V., Donghua M., Van L.K. (2005). Patterns of HIV prevalence among injecting drug users in the cross-border area of Lang Son Province, Vietnam, and Ning Ming County, Guangxi Province, China. BMC Public Health.

[11] Lang Son People’s Committee (2015). Plan on Ensuring Financial Support for HIV/AIDS Prevention and Control Activities in Lang Son Province for the 2015–2020 Period. https://thuvienphapluat.vn/van-ban/Tai-chinh-nha-nuoc/Ke-hoach-75-KH-UBND-2015-bao-dam-tai-chinh-phong-chong-HIVAIDS-Lang-Son-giai-doan-2015-2020-287209.aspx.

[12] Stevenson M., Hung D.V., Hoang T.H., Mai Anh L., Nguyen Thi Hong T., Le Tran N. (2015). Evaluation of the Vietnamese A6 Mortality Reporting System: All-Cause Mortality. Asia Pac. J. Public Health.

[13] Stevenson M.R., Ngoan le T., Hung D.V., Huong Tu N.T., Mai A.L., Ivers R.Q., Huong H.T. (2012). Evaluation of the Vietnamese A6 mortality reporting system: Injury as a cause of death. Inj. Prev..

[14] Ngoan le T., Lua N.T., Hang L.T. (2007). Cancer mortality pattern in Viet Nam. Asian Pac. J. Cancer Prev..

[15] (2006). Cancer Mortality in a Hanoi Population, Viet Nam, 1996-2005. Asian Pac. J. Cancer Prev..

[16] Ahmad O.B., Boschi Pinto C., Lopez A.D. (2001). Age Standardization of Rates: A New WHO Standard.

[17] Aldaz P., Moreno-Iribas C., Egüés N., Irisarri F., Floristan Y., Sola-Boneta J., Martínez-Artola V., Sagredo M., Castilla J. (2011). Mortality by causes in HIV-infected adults: Comparison with the general population. BMC Public Health.

[18] Zhao Y., Wu Z., McGoogan J.M., Shi C.X., Li A., Dou Z., Ma Y., Qin Q., Brookmeyer R., Detels R. (2018). Immediate Antiretroviral Therapy Decreases Mortality Among Patients With High CD4 Counts in China: A Nationwide, Retrospective Cohort Study. Clin. Infect. Dis..

[19] Trickey A., McGinnis K., Gill M.J., Abgrall S., Berenguer J., Wyen C., Hessamfar M., Reiss P., Kusejko K., Silverberg M.J. (2024). Longitudinal trends in causes of death among adults with HIV on antiretroviral therapy in Europe and North America from 1996 to 2020: A collaboration of cohort studies. Lancet HIV.

[20] Ashraf H., Nadeem A., Ashfaq H., Fatima T., Ahmed S., Nadeem Z.A., Saleh A. (2024). Disparities in mortality trends of adults with HIV in the USA: A comprehensive examination across 2 decades. Medicine.

[21] Coelho L., Grinsztejn B., Castilho J.L., De Boni R., Quintana M.S., Campos D.P., Ribeiro S.R., Pacheco A.G., Veloso V.G., Luz P.M. (2016). Mortality in HIV-infected women, heterosexual men, and men who have sex with men in Rio de Janeiro, Brazil: An observational cohort study. Lancet HIV.

[22] Gao D., Zou Z., Dong B., Zhang W., Chen T., Cui W., Ma Y. (2019). Secular trends in HIV/AIDS mortality in China from 1990 to 2016: Gender disparities. PLoS ONE.

[23] Gheibi Z., Dianatinasab M., Haghparast A., Mirzazadeh A., Fararouei M. (2020). Gender difference in all-cause mortality of people living with HIV in Iran: Findings from a 20-year cohort study. HIV Med..

[24] Girum T., Wasie A., Lentiro K., Muktar E., Shumbej T., Difer M., Shegaze M., Worku A. (2018). Gender disparity in epidemiological trend of HIV/AIDS infection and treatment in Ethiopia. Arch. Public Health.

[25] Frank T.D., Carter A., Jahagirdar D., Biehl M.H., Douwes-Schultz D., Larson S.L., Arora M., Dwyer-Lindgren L., Steuben K.M., Abbastabar H. (2019). Global, regional, and national incidence, prevalence, and mortality of HIV, 1980–2017, and forecasts to 2030, for 195 countries and territories: A systematic analysis for the Global Burden of Diseases, Injuries, and Risk Factors Study 2017. Lancet HIV.

[26] Orza L., Bass E., Bell E., Crone E.T., Damji N., Dilmitis S., Tremlett L., Aidarus N., Stevenson J., Bensaid S. (2017). In Women’s Eyes: Key Barriers to Women’s Access to HIV Treatment and a Rights-Based Approach to their Sustained Well-Being. Health Hum. Rights.

[27] Toth M., Messer L.C., Quinlivan E.B. (2013). Barriers to HIV care for women of color living in the Southeastern US are associated with physical symptoms, social environment, and self-determination. AIDS Patient Care STDS.

[28] Park E., Stockman J.K., Thrift B., Nicole A., Smith L.R. (2020). Structural Barriers to Women’s Sustained Engagement in HIV Care in Southern California. AIDS Behav..

[29] Liu Y., Osborn C.Y., Qian H.Z., Yin L., Xiao D., Ruan Y., Simoni J.M., Zhang X., Shao Y., Vermund S.H. (2016). Barriers and Facilitators of Linkage to and Engagement in HIV Care Among HIV-Positive Men Who Have Sex with Men in China: A Qualitative Study. AIDS Patient Care STDS.

